# Polysaccharides in Selenium-Enriched Tea: Extraction Performance under Innovative Technologies and Antioxidant Activities

**DOI:** 10.3390/foods11172545

**Published:** 2022-08-23

**Authors:** Weilan Gao, Na Zhang, Shuyi Li, Shuyao Li, Shiyu Zhu, Xin Cong, Shuiyuan Cheng, Francisco J. Barba, Zhenzhou Zhu

**Affiliations:** 1 College of Food Science and Engineering, Wuhan Polytechnic University, Wuhan 430023, China; 2 National R&D Center for Se-rich Agricultural Products Processing, Hubei Engineering Research Center for Deep Processing of Green Se-rich Agricultural Products, School of Modern Industry for Selenium Science and Engineering, Wuhan Polytechnic University, Wuhan 430023, China; 3 Enshi Se-Run Material Engineering Technology Co., Ltd., Enshi 445000, China; 4 Preventive Medicine and Public Health, Food Science, Toxicology and Forensic Medicine Department, Faculty of Pharmacy, Universitat de València, Avda. Vicent Andrés Estellés, 46100 Burjassot, València, Spain

**Keywords:** pulsed electric fields, selenium-enriched tea, selenium, polysaccharide, antioxidant activity

## Abstract

Pulsed electric fields (PEF) and ultrasonic-assisted extraction (UE) were applied to improve the extraction performance of selenium-enriched tea polysaccharides (Se−TPSs) in mild conditions. Two combined extraction processes were investigated: (1) PEF strength at 10 kV/cm followed by conventional extraction (CE) at 50 °C for 60 min and (2) PEF+UE (PEF strength at 10 kV/cm followed by UE at 400 W for 60 min). The optimal extraction yields, and energy consumption rates were obtained at 36.86% and 41.53% and 78.78 kJ/mg and 133.91 kJ/mg, respectively. The Se−TPSs were analyzed and characterized by GPC, UV, and FT-IR, which evidenced the structural stability of the Se−TPSs during the extraction processes. It was found that PEF and UE could reduce the particle size diameter of the Se−TPS extract, as well as the proportion of uronic acid. Moreover, PEF could increase the selenium content in the Se−TPS extract by 160.14% due to a lower extraction temperature compared to conventional extraction. The antioxidant activities of the Se−TPSs in vitro were investigated using OH, $O_{2}^{-}$, and ${\mathrm{ABTS}}^{+}$ scavenging experiments, as well as a total antioxidant ability evaluation. It was found that the antioxidant activity of the Se−TPSs obtained using PEF_2_+CE_2_ was relatively high due to the potential synergistic effect between the selenium and polysaccharides. Based on these results, we speculate that PEF_2_+CE_2_ was the best extraction process for the Se−TPSs. Furthermore, this research indicates the application of selenium-enriched tea for functional food production.

## 1. Introduction

Tea is one of the most popular beverages in the world due to its health benefits. Tea contains a great variety of bioactive compounds, such as polyphenols, polysaccharides, alkaloids, free amino acids, aromas, and saponins. Recent studies have shown that water-soluble tea polysaccharides possess many bioactive effects, such as antioxidant [1,2], anti-inflammatory [3], anti-cancer [4,5], and anti-sepsis effects [6]. They can also lower blood glucose [7,8,9] and they have immune-regulatory [10] functions. Selenium-enriched green tea, in which the selenium content is 0.25–4 mg/kg, exhibits superior antioxidant capacity over native green tea [11,12].

Conventionally, tea polysaccharides are extracted with hot water for 1–3 h after pretreatment with ethanol [13,14]. To overcome the disadvantages of a long extraction time and high energy consumption, various experiments have been carried out with the assistance of innovative technologies, such as microwave, ultrasonic, and pulsed electric fields (PEF). Ultrasonic and PEF-assisted extraction have been proven to be effective at improving the extraction rate of tea polysaccharides [15] by breaking the tea leaf cell wall and improving the mass transfer during extraction [16]. The studies on selenium-enriched tea polysaccharides (Se−TPSs) have been mainly focused on the activity and structure of polysaccharides [17]. However, investigating the effects of the extraction condition on Se−TPS, especially its impact on selenium content, has rarely been explored [18,19]. Although PEF and ultrasonic treatments are often applied to enhance the extraction process, the comparison of these non-thermal technologies, as well as their combined effect on the Se, is still lacking, which greatly limits the recovery of functional products from selenium-enriched tea [18].

In this study, pulsed electric field (PEF) and ultrasonic extraction (UE) were introduced to improve the Se−TPS extraction yield from selenium-enriched tea at mild conditions and low specific energy levels. The effects of PEF and UE on the selenium content in Se−TPSs were investigated. The obtained Se−TPSs were analyzed and characterized by GPC, UV, and FT-IR to explore the structural stability and differences. The antioxidant activities of Se−TPSs with different selenium contents were evaluated to explore the synergistic effect between selenium and tea polysaccharides. This study could lead to a new valorization method for selenium-enriched tea.

## 2. Materials and Methods

### 2.1. Material, Reagents, and Pretreatment

Selenium-enriched green tea was purchased from Qin-Xiyuan Selenium Tea Company (Enshi, Hubei, China). It was naturally grown in the selenium-rich area of Enshi without the exogenous supplement of selenium fertilizer. The tea leaves were ground into powder until the sample could pass through an 80-mesh sieve, and then stored at −20 °C for future study. Dextran with standard weight-average molecular weights (Mw 9750, 13,050, 36,800, 64,650, and 135,350 Da) was purchased from the China National Institute for Food and Drug Control. Standard monosaccharides, including fucose (Fuc), rhamnose (Rha), arabinose (Ara), galactose (Gal), glucose (Glc), xylose (Xyl), mannose (Man), fructose (Fru), ribose (Rib), galacturonic acid (GalA), glucuronic acid (GlcA), galacturonic acid (GalA), and mannuronic acid (ManA), were purchased from BoRui Saccharide Biotech Co., Ltd. (Yangzhou, Jiangsu, China). Hydroxyl radical (OH), superoxide anion radical ($O_{2}^{-}$), and 2,2′-azinobis (3-ethylbenzothiazoline-6-sulphonic acid) radical (${\mathrm{ABTS}}^{+}$) scavenging activity assay kits were purchased from Solaibao Technology Co., Ltd. (Beijing, China). All other reagents were analytical or chromatographic.

The pretreatment of the tea powder was performed following a previously reported procedure with slight modifications [20]. Ethanol (95%) was added to the tea powder at a ratio of 10:1 (*v*/*w*), and then the solution was stirred for 4 h at room temperature to decolorize and remove the small molecule components, after which the solution was centrifuged at 5000 rpm for 30 min at 25 °C. This procedure was repeated for more than five times until the supernatant was colorless and transparent. Then the precipitate was dried at 45 °C for future study.

### 2.2. Selenium-Enriched Tea Polysaccharide (Se−TPS) Extraction

**Figure 1:**
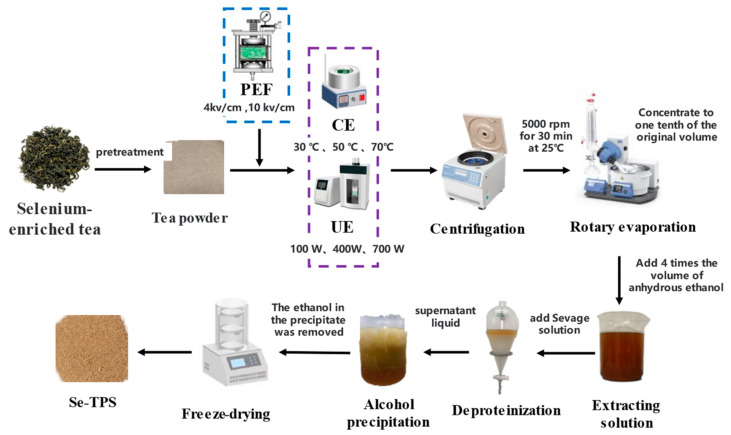
Extraction process of Se−TPSs.

The extraction of Se−TPSs from selenium-enriched green tea was carried out based on our previous studies with slight modifications using four different methods: (1) conventional extraction (CE) using water as a solvent, (2) ultrasonic extraction (UE), (3) pulsed electric field (PEF) treatment followed by a conventional extraction (PEF+CE), and (4) PEF treatment followed by UE (PEF+UE) [21]. The extraction process of Se−TPSs is shown in Figure 1.

For each extraction experiment, 5.0 g of pretreatment Se-enriched green tea powder was used as the raw material. The parameters, including the extraction temperature (30–70 °C), liquid-to-solid ratio (15–40), and time (10–120 min), were investigated during the CE process. The effect of the ultrasonic power (100–700 W) was investigated in the UE process. For the PEF+CE process, the PEF strength (4 kV/cm–10 kV/cm) was investigated at a half-peak width of 54 μs, frequency of 1.04 kHz, and treatment time of 1.80 ms. The detailed information for each extraction test is listed in Table S1. After each extraction experiment, the solution was centrifuged at 5000 rpm for 30 min at 25 °C, and the supernatant was collected for future study.

### 2.3. Se−TPS Purification

As shown in Figure 1, the resulting supernatant from the extraction was collected, concentrated to one-tenth of its original volume under vacuum concentration at 45 °C, and then treated with Sevage reagent to remove the protein, followed by the addition of the triploid Sevage solution (chloroform/n-butanol = 4:1). After shaking for 30 min, the precipitate was discarded by centrifugation. The above operation was repeated until no flocculent precipitation occurred in order to ensure complete protein removal in the extract. The supernatant was transferred to the beaker, and anhydrous ethanol at a volume four times greater was added (to make the ethanol content in the solution reach 80%) while stirring. The solution was kept at 4 °C for 12 h to precipitate the polysaccharides. A small amount of ultra-pure water was used to dissolve the precipitate, and 45 °C rotary evaporation was used to remove the ethanol in the polysaccharide precipitate. After freeze-drying for 48 h, the crude polysaccharides of the green tea were obtained. The Se−TPSs from the raw material were determined by the phenol–sulfuric acid method, using glucose as the standard [22].

The extraction yield for the extraction process was calculated according to Equation (1), and the specific energy consumption rates for the CE, UE, and PEF processes were calculated according to Equations (2), (3), and (4), respectively.

The extraction rates and specific energy consumption rates of the extraction process were calculated as follows:

(1) $$\mathrm{Extraction}\;\mathrm{yield}=\frac{\mathrm{Se}-\mathrm{TPS}\;\mathrm{in}\;\mathrm{the}\;\mathrm{extract}}{\mathrm{TPS}\;\mathrm{of}\;\mathrm{the}\;\mathrm{Selenium}-\mathrm{enriched}\;\mathrm{green}\;\mathrm{tea}}$$

This is the equation for the specific energy consumption of CE:

(2) $$Q_{1}=\frac{m\times c\times \left(T_{1}-T_{2}\right)}{V\times M}$$

This is the equation for the specific energy consumption of UE:

(3) $$Q_{2}=\frac{P\times t}{V\times M}$$

This is the equation for the specific energy consumption of PEF:

(4) $$Q_{3}=E^{2}\times \frac{f\times V_{0}}{S\times M}\times W_{P}\times n\times \delta$$

where Q is the specific energy consumption, kJ/mg; m is the mass of the liquid heated in the water bath, g; c is the specific heat capacity of the water, 4.18 kJ/kg·°C; T_1_ is the heating temperature, °C; T_2_ is the initial temperature, °C; V is the volume of extraction, mL; M is the content of the Se−TPS of extraction, mg/mL; P is the power of UE, W; t is the time of the treatment, s; E is the electric field strength, kV/cm; f is the pulse frequency, kHz; V_0_ is the treatment chamber volume of PEF, mL; S is the injection flow rate of PEF, mL/s; W_p_ is the pulse width of PEF, μs; n is the processing time of PEF; δ is the electrical conductivity of the extracting solution, S/m.

### 2.4. Physicochemical Properties Analysis and Structural Characterization

#### 2.4.1. Component Analysis

The neutral sugar content of the Se−TPSs was determined by the phenol–sulfuric acid method, using glucose as the standard [22]. The uronic acid content of the Se−TPSs was determined by the m-hydroxybiphenyl method, using galacturonic acid as the standard [23]. The reducing sugar content of the Se−TPSs was determined by the dinitrosalicylic acid method, using glucose as the standard [24]. The protein content was measured by the Bradford method, using bovine serum albumin as the standard [25]. The Folin–Ciocalteau colorimetric method was used to determine the total polyphenols in the Se−TPS samples [26]. The total selenium content was measured by atomic fluorescence [21].

#### 2.4.2. Monosaccharide Composition Analysis

The composition and content of monosaccharides in the Se−TPSs were determined by ion chromatography. Firstly, 5 mg of the sample was weighed accurately and placed in ampoules, adding 2 mL of 3 M trifluoroacetic acid. It was then hydrolyzed for 2 h at 120 °C. Once finished, the resultant was transferred into the tube and dried by nitrogen blowing. Subsequently, 10 mL of water was added for the vortex, of which 40 µL of the solution was taken and diluted to 1 mL. Then, the diluent was centrifuged at 12,000 rpm for 5 min, and the supernatant was injected for further analysis. Chromatographic column: Dionex Carbopac TMPA20 (3 × 150 mm). Moving phase: A: H_2_O, B: 15 mM NaOH, C: 15 mM NaOH, and 100 mM NaOAC. Low rate: 0.3 mL/min. Injection volume: 5 µL. Column temperature: 30 °C. Detector: electrochemical detector.

#### 2.4.3. Molecular Mass Analysis

The molecular weight of the Se−TPSs was determined by gel permeation chromatography (GPC) in liquid chromatography (Agilent 1100) with a differential refractive index detector (RID) and a TSK GEL G 4000 PWxL gel column (300 × 7.8 mm, 10 µm). The mobile phase was 0.1 mol/L sodium nitrate aqueous solution, the flow rate was 1.0 mL/min, the column temperature was 35 °C, and the injection volume was 20 mL (1 mg/mL, *w*/*v*). According to the retention time of different Mw rates (9750, 13,050, 36,800, 64,650, and 135,350 Da) of the dextran molecular weight standard (National Institute for Food and Drug Control), the standard curve was obtained and the molecular weight of the Se−TPSs was calculated.

#### 2.4.4. Determination of Particle Size Distribution

The particle size and distribution of the Se−TPS samples were determined using a Malvern laser particle size analyzer. The sample was prepared as a 10 mg/mL solution with a refractive index of 1.565 and an absorptivity of 0.01. The dispersant was water with a refractive index of 1.33.

#### 2.4.5. Ultraviolet Spectral Analysis (UV)

A small amount of the Se−TPS sample was prepared as a sample solution (1 mg/mL), and an A580 AOELAB UV spectrophotometer (Aoe Instruments Shanghai Co., Ltd., Shanghai, China) was used for full scanning in the wavelength range of 200–400 nm.

#### 2.4.6. Fourier Transform Infrared Spectroscopy (FT-IR)

The infrared spectrum analysis of the Se−TPS samples was carried out using the potassium bromide tablet method. The dry samples were mixed and ground evenly with 1 mg and 100 mg of potassium bromide and then made into transparent tablets. A Fourier transform infrared spectrometer was used to conduct spectral scanning in the range of 500–4000 cm^−1^.

### 2.5. Analysis of Antioxidant Activity

The antioxidant ability of the Se−TPS extract was analyzed by measuring its scavenging capacity of hydroxyl radical (OH) [27], superoxide anion radical ($O_{2}^{-}$) [20], 2,2′-azinobis(3-ethylbenzothiazoline-6-sulphonic acid) radical (${\mathrm{ABTS}}^{+}$) [28], and total antioxidant capacity (T-AOC), respectively [29].

### 2.6. Statistical Analysis

All data were represented as the mean ± standard deviation (*n* = 3), and the data were analyzed with SPSS 22.0 (IBM, Chicago, IL, USA), GraphPad Prism 8 (Software, San Diego, CA, USA), and Excel 2010 (Microsoft, Washington, D.C., USA) software. Statistical analysis was carried out using ANOVA plus post hoc Duncan’s test. The error bars in the figures represent the standard error. Different lowercase letters within a specific concentration note significant differences (*p* < 0.05). Pearson’s correlation tests were conducted to determine the correlations between the variables.

## 3. Results and Discussion

### 3.1. Se−TPS Yield and Specific Energy Consumption of Different Extraction Methods

The components in the Se-enriched green tea were examined and are listed in Table S2. The contents of protein, tea polyphenol, caffeine, free amino acid, total sugar, and selenium were 28.70 ± 0.60%, 22.61 ± 0.46%, 4.36 ± 0.11%, 4.97 ± 0.09%, 13.69 ± 0.88%, and 2.16 ± 0.08 mg/kg, respectively, which meet the standards of Se-enriched tea (Se content at 0.25 mg/kg~4.0 mg/kg).

To optimize the parameters of conventional extraction, the Brix value was used to express the Se−TPS extraction rate, which has a certain correlation with the sugar content in the extract [30]. Because of its advantages, such as convenience and efficiency, saccharometers have been widely used for the extraction of some carbohydrate-based crops [30]. As shown in Figure S1, the highest soluble solid content (51.81%) was achieved at 70 °C, while the content of soluble solids decreased with the increase in temperature, which may have been due to the degradation of the soluble solids [31]. At the initial stage, the extraction time significantly influenced the extraction yield; however, the soluble solids content reached equilibrium after 60 min. An increase in the liquid/solid ratio from 15:1 to 30:1 resulted in the rapid augmentation of the extraction yield of the Se−TPSs, reaching a peak of (52.41%) due to the intensified mass transfer of solutes at a lower concentration. An optimal liquid/solid ratio of 30:1 was also demonstrated in the ultrasound-assisted valorization of tea polysaccharides previously [15].

**Figure 2:**
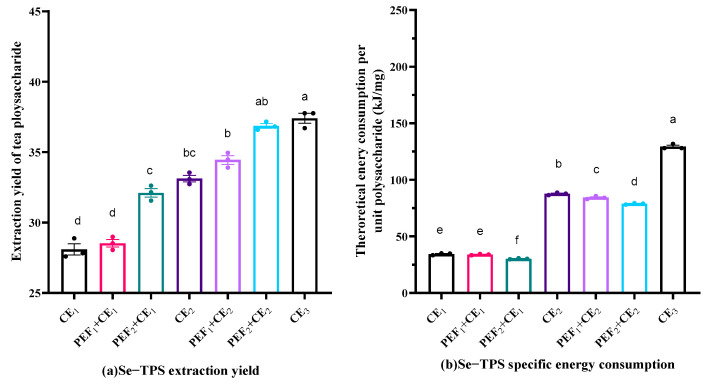
Se−TPS extraction yield (**a**) and specific energy consumption (**b**) in CE and PE + CE processes. Different letters above the columns represent extraction yield and energy consumption differ significantly (*p* < 0.05).

**Figure 3:**
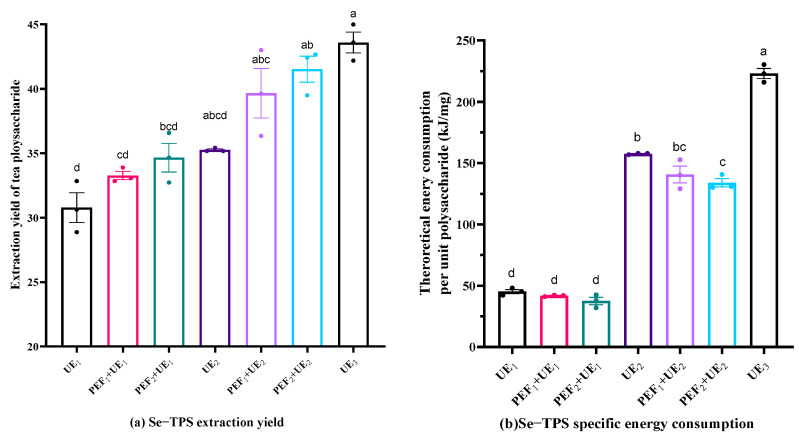
Se−TPS extraction yield (**a**) and specific energy consumption (**b**) in UE and PE + UE processes. Different letters above the columns represent extraction yield and energy consumption differ significantly (*p* < 0.05).

The effects of the parameters on the Se−TPS extraction yield and specific energy consumption are presented in Figure 2 and Figure 3. As shown in Figure 2, the Se−TPS extraction yield and specific energy consumption rate for CE_3_ (conventional extraction at 70 °C for 60 min) were 37.40% and 128.08 kJ/mg, respectively. The application of the PEF treatment significantly improved the extraction performance. For example, the Se−TPS extraction yield and the specific energy consumption for the PEF_2_+CE_2_ process (pulsed electric field strength of 10 kV/cm pretreatment, followed by conventional extraction at 50 °C for 60 min) were 36.86% and 78.78 kJ/mg, respectively. With the assistance of the PEF treatment, a comparable extraction yield was achieved with a much lower energy consumption rate.

The Se−TPS extraction performance under UE is shown in Figure 3. As expected, an increase in the ultrasonic power resulted in a higher Se−TPS extraction yield (Figure 3a) but also led to a higher specific energy consumption rate (Figure 3b). The combination of PEF and UE seems to have been more promising for obtaining an ideal Se−TPS extraction yield at a relatively low specific energy consumption rate. As indicated in Figure 3a, the Se−TPS extraction yield and specific energy consumption rate for PEF_2_+UE_2_ (pulsed electric field strength of 10 kV/cm pretreatment, followed by ultrasonic extraction at 400 W for 60 min) were 41.53% and 133.91 kJ/mg, respectively. The yield and specific energy consumption rate for UE_3_ (ultrasonic extraction at 700 W for 60 min) were 43.59% and 223.04 kJ/mg, respectively.

### 3.2. Physicochemical Properties of Se−TPS

#### 3.2.1. Component Analysis

To investigate the effects of PEF and ultrasound on the composition of Se−TPS extract, a chemical analysis was carried out (Table 1). It was found that the neutral sugar content of Se−TPS increased after the physical field treatment (PEF_2_+UE_2_ (35.76%) > PEF_2_+CE_2_ (28.64%) > CE_1_ (25.06%)), while the content of uronic acid decreased (CE_1_ (32.21%) > PEF_2_+CE_2_ (23.41%) > PEF_2_+UE_2_ (23.03%)). This result implies that a physical field treatment could change the contents of neutral sugar and uronic acid in Se−TPSs, and the increase in the neutral sugar content may be due to the degradation of uronic acid [15]. A composition analysis found that the neutral sugars and uronic acid were more abundant in Se−TPSs, both exceeding 20%, followed by tea polyphenols and tea protein, accounting for about 10%, and the contents of reducing sugars and selenium were relatively lower. A protein content analysis revealed that Se−TPSs might be a type of glycoprotein, and the physical field treatment caused glycosidic bond hydrolysis, thereby influencing the protein content. The PEF treatment resulted in higher total selenium (3.85 ± 0.28 mg/kg) in the Se−TPS but, unexpectedly, a combination of PEF and ultrasonic extraction led to a lower selenium content (1.93 ± 0.06 mg/kg). To some extent, the uncertainty of varying ultrasonic parameters from the aspect of the output enhancement of active ingredients from certain bio-sources has also been reported previously [32].

#### 3.2.2. Monosaccharide Composition

The monosaccharide composition of Se−TPS was further studied using ion chromatography. Figure S2 is the ion chromatogram of Se−TPS, in which the peak of sodium hydroxide appears at 2 min and the peak of sodium acetate appears at 40 min. Table 2 summarizes the mole ratio of the monosaccharides in Se−TPSs. The main monosaccharides in the Se−TPSs were found as Rha, Ara, Gal, Glc, Xyl, Man, and GalA. Other monosaccharides were not detected, or their content was below the detection limit. Wang et al. (2012) demonstrated that oolong tea polysaccharides consist of seven monosaccharides, among which Glc, Gal, and Ara were the dominant compounds [33]. The results suggest that the PEF and UE treatments did not alter the monosaccharide composition of the extracted Se−TPSs but changed the molar ratio slightly, which is consistent with the research results of Dou et al. [34].

#### 3.2.3. Physicochemical Characterization of Se−TPSs

**Figure 4:**
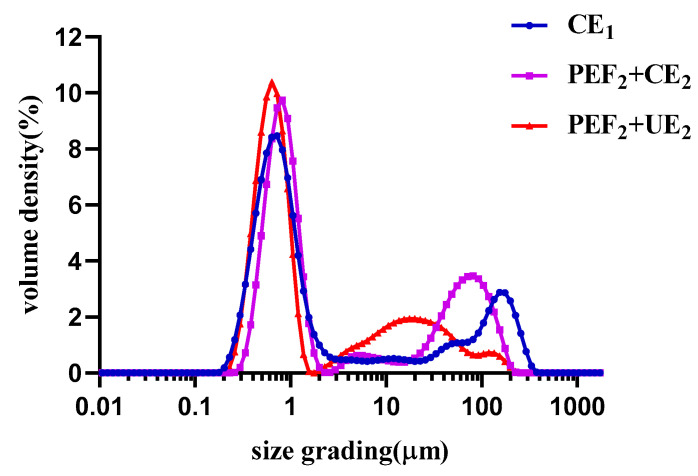
The particle size distribution of Se−TPSs obtained using different extraction methods.

The particle size distribution of Se−TPSs is shown in Figure 4 and Table 3. The average volume diameter D[4,3] of the Se−TPSs obtained using different extraction methods varied greatly, with CE_1_ (34.7 μm) > PEF_2_+CE_2_ (26.5 μm) > PEF_2_+UE_2_ (11.8 μm), which may be attributed to the damage of the Se−TPSs under the PEF or ultrasonic treatments. It appears that the stronger physical field treatment resulted in increased breakage of the extract particles, leading to a decrease in sequence [35]. Moreover, it was evident that D × 10 and D × 50 both presented trends of PEF_2_+CE_2_ > CE_1_ > PEF_2_+UE_2_. Therefore, it was speculated that PEF may have caused the aggregation of small Se−TPS molecules with charges [36], which contributed to larger values of 10% and 50% of the Se−TPS particles obtained using PEF_2_+CE_2_. Ultrasound generates cavitation as well as a mechanical effect, resulting in smaller particles [15]. Thus, the Se−TPSs obtained using PEF_2_+UE_2_ showed no significant change compared to the Se−TPS obtained using CE_1_, and the variation trend of D × 90 is inconsistent with D[4,3]. Wang et al. (2021) studied the influence of ultrasonic irradiation on the particle size of tea polysaccharides and obtained similar results [20].

**Figure 5:**
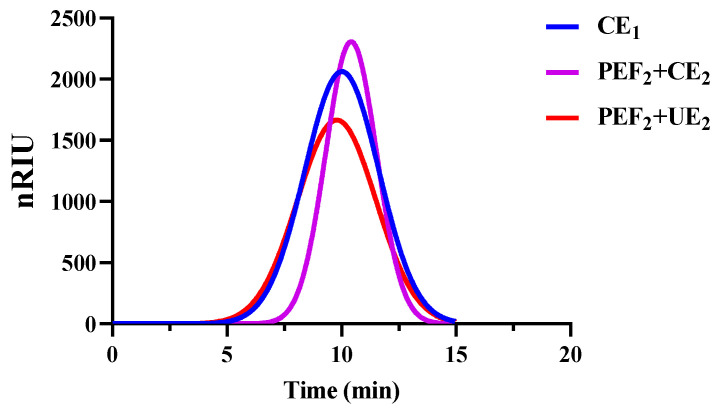
Elution curves of Se−TPSs using gel permeation chromatography.

The molecular weight analyses of the Se−TPSs are included in Figure 5 and Table 4. The Mw/Mn values of the Se−TPSs obtained using CE_1_, PEF_2_+CE_2_, and PEF_2_+UE_2_ were 6.15, 7.99, and 5.42, respectively, which is consistent with the D × 10 and D × 50 particle sizes. This further suggests that PEF may have led to Se−TPSs with a low degree of aggregation and increased the number average molecular weight Mn and weight average molecular weight Mw, resulting in the long and short chains of Se−TPSs being unevenly distributed and poorly dispersed. The ultrasonic treatment destroyed the sugar chains of the Se−TPSs, resulting in relatively better dispersion and a lower molecular weight of the Se−TPSs obtained using PEF_2_+UE_2_ compared to the Se−TPSs obtained using PEF_2_+CE_2_. These results also demonstrate the good effect of the physical field treatment on the molecular weight and dispersion of Se−TPSs (especially Se−TPSs with high molecular weight) [37].

**Figure 6:**
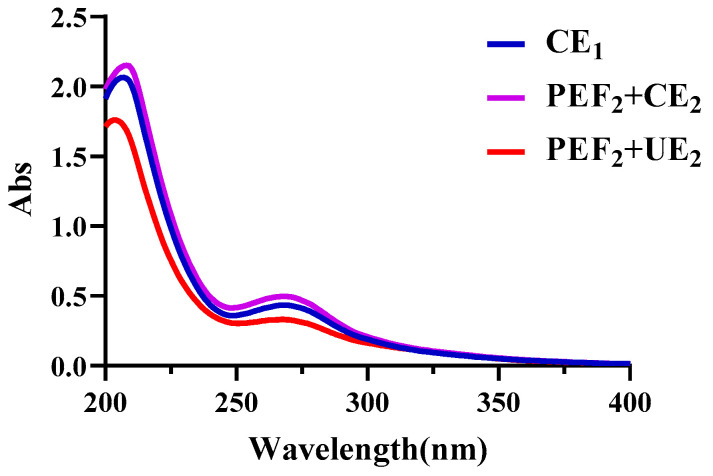
The UV−vis spectra of Se−TPSs obtained using different extraction methods and studied in the range of 200~400 nm.

To determine the effects of the physical field treatment on the structure of Se−TPSs, the UV spectra of the polysaccharide samples were recorded and are shown in Figure 6. It was found that different Se−TPSs obtained using CE_1_, PEF_2_+CE_2_, and PEF_2_+UE_2_ all had a maximum absorption peak at 267 nm, indicating the existence of nucleic acid proteins in Se−TPSs. The spectral absorption peaks of CE_1_ and PEF_2_+CE_2_ at 267 nm were higher than those of PEF_2_+UE_2_, suggesting that the protein contents of the Se−TPSs obtained using CE_1_ and PEF_2_+CE_2_ were more abundant than those obtained using PEF_2_+UE_2_, which is consistent with the results collected in the composition analysis shown in Table 1.

**Figure 7:**
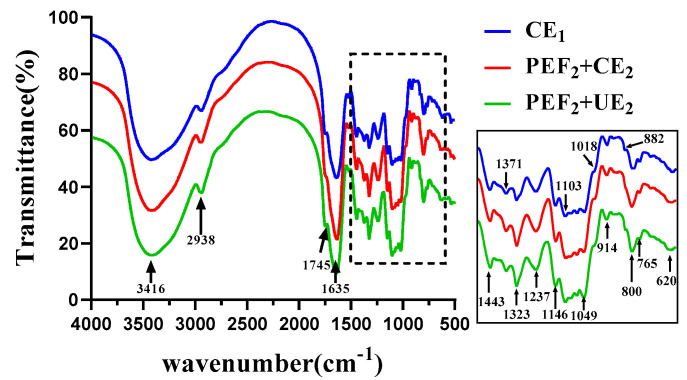
The FT−IR spectra of Se−TPSs obtained using different extraction methods and studied in the range of 200~400 nm.

To clarify the structural details of the Se−TPSs, Fourier transform infrared spectroscopy (FT−IR) analysis was performed and the results can be seen in Figure 7. The wide and strong absorption peak at around 3416 cm^−1^ was caused by the O-H stretching vibration of the hydroxyl group [38]. The absorption peaks at around 2938 cm^−1^ and 1443 cm^−1^ were caused by the C-H stretching and bending vibrations of the alkyl groups, respectively. Both the hydroxyl and alkyl groups are characteristic units of polysaccharides, the occurrence of which evidenced the main structure of Se−TPSs [39]. The peaks at 1745 cm^−1^ and 1635 cm^−1^ refer to the typical vibrations of C=O stretching in the acetyl group [34,40]. The absorption peak at 1323 cm^−1^ is attributed to the carboxylic acid group, indicating the presence of aldehyde acids in the polysaccharide sample [41]. The area between 800 and 1200 cm^−1^ can be observed as the fingerprint region of sugar, which can determine the type and configuration of sugar [42]. The absorption peaks at 1103 cm^−1^, 1018 cm^−1^, 914 cm^−1^, and 882 cm^−1^ are typical peaks of angular oscillations of the hydroxyl O-H on the pyran ring, possibly due to the presence of arabinose, rhamnose, galactose, and mannose [20]. Furthermore, the intensity differences in the absorption peaks between different Se−TPS samples reflect alterations in the chemical compositions of the polysaccharides after various treatments to a certain extent. The absorption peak near 765 cm^−1^ is attributed to the four adjacent hydrogen atoms in the benzene ring, revealing the presence of aromatic amino acids. The absorption peak at 620 cm^−1^ indicates the presence of phenolic compounds in tea polysaccharides [43]. Above all, Se−TPS extract could be identified as complex polysaccharides containing proteins and uronic acids. The PEF and UE treatments could cause the rupture of the glycosidic bonds of Se−TPSs, but the main functional groups were not significantly changed [44].

### 3.3. In Vitro Antioxidant Activities of Se−TPSs

**Figure 8:**
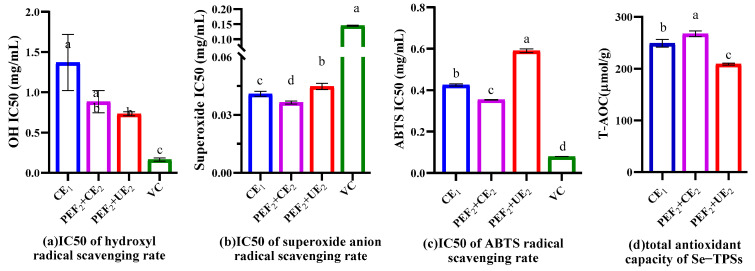
IC50 of hydroxyl radical scavenging rate (**a**), superoxide anion radical scavenging rate (**b**), ABTS radical scavenging rate (**c**), and total antioxidant capacity of Se−TPSs (T-AOC) (**d**). Different letters above the columns represent siginificantly different antioxidant activities (*p* < 0.05).

Free radical-induced oxidative stress is one of the most important underlying mechanisms resulting in different diseases, including cancer, neurodegenerative disorders, and inflammatory diseases [45]. Therefore, the antioxidant activities of Se−TPSs in vitro were investigated using OH, $O_{2}^{-}$, and ${\mathrm{ABTS}}^{+}$ scavenging experiments, as well as a total antioxidant ability evaluation (shown in Figure S3 and Figure 8).

In this study, the free radical scavenging ability of Se−TPSs was presented in Figure S3, ranging from 0~2 mg/mL. It was found that all Se−TPSs exhibited strong radical scavenging capacity in a concentration-dependent manner. As indicated in Figure 8a,c, the scavenging ability of Se−TPSs on OH, and ${\mathrm{ABTS}}^{+}$ was weaker than that of vitamin C (V_C_). However, the Se−TPSs obtained using different extraction methods all possessed higher $O_{2}^{-}$ scavenging activity than V_C_, as suggested in Figure 8b. The IC_50_ values of the Se−TPSs obtained using PEF_2_+CE_2_ on the scavenging ability for OH, $O_{2}^{-},$ and ${\mathrm{ABTS}}^{+}$ were 0.745, 0.0355, and 0.354 mg/mL, respectively, which are lower than those of the Se−TPSs obtained using CE_1_ (IC_50_ values of 1.37, 0.0397, and 0.422 mg/mL, respectively). That is to say, the in vitro antioxidant activities of the Se−TPSs obtained using PEF_2_+CE_2_ were significantly better, which was probably due to its higher selenium content, as shown in Table 1. As reported, selenium has been demonstrated in many reports to exhibit excellent antioxidant activity [46]. However, the Se−TPSs obtained using PEF_2_+UE_2_ had larger IC_50_ values than CE_1_ on scavenging $O_{2}^{-}$ and ${\mathrm{ABTS}}^{+}$ and the lowest T-AOC, indicating that the bioactivities of tea polysaccharides treated with PEF_2_+UE_2_ were inhibited [35]. This phenomenon may be attributed to a lower uronic acid content in the polysaccharide extract obtained using PEF_2_+UE_2_. Some studies have proven that the level of uronic acid is positively correlated with the free radical scavenging ability [47]. Thus, this research further demonstrates that the antioxidant effects of Se−TPSs obtained using different extraction methods vary to a notable degree.

**Figure 9:**
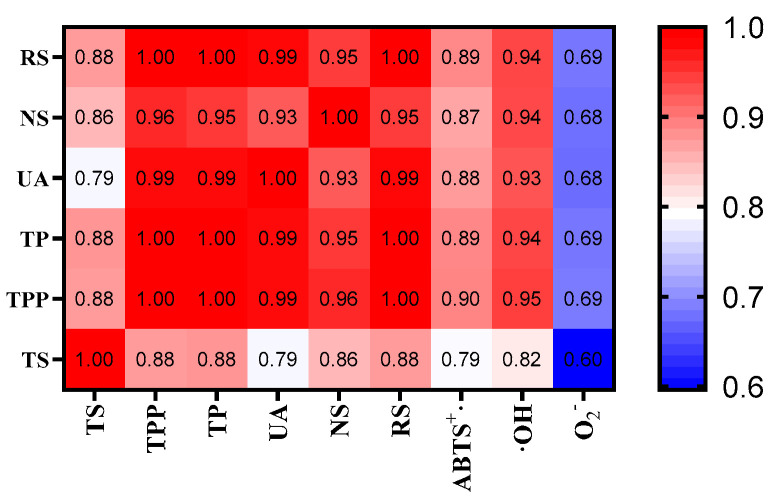
Heat map analysis of the correlation between chemical composition and antioxidative activity. Abbreviations: NS: neutral sugar, UA: uronic acid, RS: reducing sugar, TP: tea protein, TPP: tea polyphenol, TS: total selenium, OH: Hydroxyl radical capacity, $O_{2}^{-}$: superoxide radical capacity, ${\mathrm{ABTS}}^{+}$: ABTS radical capacity, and T−AOC: total antioxidant capacity. The data in the figure represent the Pearson correlation coefficient.

The correlation between the chemical composition and bioactive properties of Se−TPSs was evaluated using heat map analysis, as presented in Figure 9 and Table S3. All the correlations showed strong significance (*p* < 0.01). There were positive correlations of the total selenium content with tea polyphenols (Pearson r = 0.88), tea protein (Pearson r = 0.88), neutral sugars (Pearson r = 0.86), and reducing sugars (Pearson r = 0.88), indicating that selenium in tea binds mainly to proteins, polysaccharides, and polyphenols [43]. Furthermore, TS with ${\mathrm{ABTS}}^{+}$ (Pearson r = 0.79), OH (Pearson r = 0.82), and $O_{2}^{-}$ (Pearson r = 0.60) radical scavenging activities also suggest varying degrees of correlation. These results illustrate that selenium plays an important role in antioxidant activity [44]. There were positive correlations of the tea polyphenols, tea protein, uronic acid, neutral sugars, and reducing sugars with the antioxidant activities, indicating that the phenolic compounds and protein content of the Se−TPSs are also involved in the antioxidant ability, which is inconsistent with other studies [45,46,47]. Se−TPSs using PEF_2_+CE_2_ showed a better antioxidant capacity, which may be related to their higher selenium content.

## 4. Conclusions

PEF and ultrasonic treatments were applied to enhance Se−TPS extraction from selenium-enriched tea planted in Enshi, China. Four kinds of extraction processes (CE, PEF+CE, UE, and PEF+UE) were carried out to investigate their effects on the Se−TPS extraction yield and specific energy consumption rate. Based on the results, it was found that PEF_2_ + CE_2_ was the most optimal for the extraction of Se−TPS. The PEF treatment at an electrical field strength of 10 kV/cm followed by extraction at 50 °C for 60 min led to a higher Se−TPS yield (36.86%) and a lower specific energy consumption rate (78.78 kJ/mg). In addition, the Se−TPSs obtained using PEF_2_+CE_2_ were determined to have a higher selenium content (3.85 ± 0.28 mg/kg) compared to those obtained using CE_1_ (1.48 ± 0.23 mg/kg) and PEF_2_+UE_2_ (1.93 ± 0.06 mg/kg) because of better selenium preservation at a lower extraction temperature. Meanwhile, the treatments of PEF and ultrasound resulted in a significant reduction of uronic acid in the extracted product. The main monosaccharides in the Se−TPSs were Rha, Ara, Gal, Glc, Xyl, Man, and GalA. A physicochemical characterization showed that the Se−TPS structure remained stable under different extraction conditions, but the physical field treatment generated smaller particle size due to the degradation of large Se−TPS particles. An antioxidant activity analysis revealed that Se−TPSs with higher selenium contents exhibited better antioxidant activity, implying a potential synergistic effect of selenium and tea polysaccharides. In conclusion, this study provides new insight into the valorization of high added-value products from selenium-enriched plants with innovative technologies.

## Figures and Tables

**Table 1:** Chemical compositions of Se−TPSs obtained using various extraction processes *.

Samples	Neutral Sugar (%)	Uronic Acid (%)	Reducing Sugar (%)	Tea Protein (%)	Tea Polyphenol (%)	Total Selenium (mg/kg)
CE_1_	25.06 ± 0.61 ^c^	32.21 ± 0.81 ^a^	0.56 ± 0.06 ^a^	8.82 ± 0.61 ^a^	12.40 ± 0.16 ^a^	1.48 ± 0.23 ^c^
PEF_2_+CE_2_	28.64 ± 0.58 ^b^	23.41 ± 0.05 ^b^	0.54 ± 0.02 ^a^	8.70 ± 0.20 ^a^	12.18 ± 0.45 ^a^	3.85 ± 0.28 ^a^
PEF_2_+UE_2_	35.76 ± 0.53 ^a^	23.03 ± 0.65 ^b^	0.45 ± 0.04 ^b^	7.21 ± 0.56 ^b^	10.91 ± 0.54 ^b^	1.93 ± 0.06 ^b^

* Each value represents the mean ± standard deviation (*n* = 3). Statistical analysis was carried out using ANOVA plus post hoc Duncan’s test. Different lowercase letters within a specific concentration differ significantly (*p* < 0.05).

**Table 2:** Monosaccharide compositions of Se−TPSs (molar ratio).

Samples	Rha	Ara	GlcN	Gal	Glc	Xyl	Man	GalA
CE_1_	0.037	0.350	0.003	0.19	0.271	0.033	0.049	0.067
PEF_2_+CE_2_	0.031	0.289	0.001	0.239	0.278	0.036	0.038	0.088
PEF_2_+UE_2_	0.036	0.287	0.001	0.255	0.265	0.048	0.031	0.077

**Table 3:** Particle size distribution of Se−TPSs *.

Samples	D[4,3] (μm)	D[3,2] (μm)	D × (10) (μm)	D × (50) (μm)	D × (90) (μm)
CE_1_	34.47 ± 0.57 ^a^	0.65 ± 0.01 ^b^	0.41 ± 0.01 ^b^	0.92 ± 0.01 ^b^	152.67 ± 0.58 ^a^
PEF_2_+CE_2_	26.50 ± 0.46 ^b^	0.88 ± 0.01 ^a^	0.54 ± 0.01 ^a^	1.09 ± 0.01 ^a^	93.43 ± 0.55 ^b^
PEF_2_+UE_2_	11.83 ± 0.15 ^a^	0.61 ± 0.01 ^c^	0.42 ± 0.01 ^b^	0.81 ± 0.01 ^c^	34.07 ± 0.49 ^c^

* Average volume diameter D[4,3], average area diameter D[3,2]. D × 10, D × 50, and D × 90, respectively, indicate that particles with smaller diameters account for 10%, 50%, and 90% of the total. Each value represents the mean ± standard deviation (*n* = 3). Statistical analysis was carried out using ANOVA plus post hoc Duncan’s test. Different lowercase letters within a specific concentration differ significantly (*p* < 0.05).

**Table 4:** The molecular weights of Se−TPSs *.

Samples	Mn (kg/mol)	Mw (kg/mol)	Mz (kg/mol)	10% (kg/mol)	30% (kg/mol)	50% (kg/mol)	70% (kg/mol)	90% (kg/mol)	Mw/Mn
CE_1_	67.32	414.2	1152	22.969	64.18	164.96	435.98	1216.1	6.15
PEF_2_+CE_2_	105.1	839.9	1755	31.556	138.87	508.33	1153.60	2246.6	7.99
PEF_2_+UE_2_	118.2	640.4	1446	39.301	136.50	339.22	775.28	1756.2	5.42

* Mn represents the number average molecular weight, Mw represents the weight average molecular weight, Mz represents the viscosity average molecular weight, Mw/Mn represents dispersion, and 10%, 30%, 50%, 70%, and 90% mean that the parts less than the molecular weight account for 10%, 30%, 50%, 70%, and 90%.

## Data Availability

The data presented in this study are available in the article.

## Supplementary Materials

The following supporting information can be downloaded at: https://www.mdpi.com/article/10.3390/foods11172545/s1, Figure S1: Influence of the time, temperature, and liquid/solid ratio of the extraction of selenium-rich tea polysaccharides from Se-rich green tea using water extraction; Figure S2: The ion chromatograms of selenium-rich tea polysaccharides obtained using different extraction methods; Figure S3: Antioxidant capacities of CE_1_, PEF_2_+CE_2_, and PEF_2_+UE_2_; Table S1: Detailed information for Se−TPS extraction tests; Table S2: Basic ingredients of selenium-rich tea; Table S3: Correlation between the chemical composition and antioxidative activity.

## Abbreviations

**Number**	**Abbreviation**	**Full Title**
1	Se−TPS	Selenium-enriched tea polysaccharide
2	UE	Ultrasonic extraction
3	PEF	Pulsed electric field
4	CE	Conventional extraction
5	IC	Ion chromatography
6	UV	Ultraviolet spectral analysis
7	FT-IR	Fourier transform infrared spectroscopy
8	VC	Vitamin C
9	OH	Hydroxyl radical capacity
10	$O_{2}^{-}$	Superoxide radical capacity
11	${\mathrm{ABTS}}^{+}$	2,2′-azinobis(3-ethylbenzothiazoline-6-sulphonic acid) radical capacity
12	T-AOC	Total antioxidant capacity
13	NS	Neutral sugar
14	UA	Uronic acid
15	RS	Reducing sugar
16	TP	Tea protein
17	TPP	Tea polyphenol
18	TS	Total selenium
