# Supplementary material for: Polysaccharides in Selenium-Enriched Tea: Extraction Performance under Innovative Technologies and Antioxidant Activities
Source: Foods. 2022 Aug 23;11(17):2545. doi: 10.3390/foods11172545 (PMC9455174; doi:10.3390/foods11172545)
Supplement: Supplementary file 1 [file foods-11-02545-s001.zip › Supplementary Materials.pdf]

Supplementary Materials

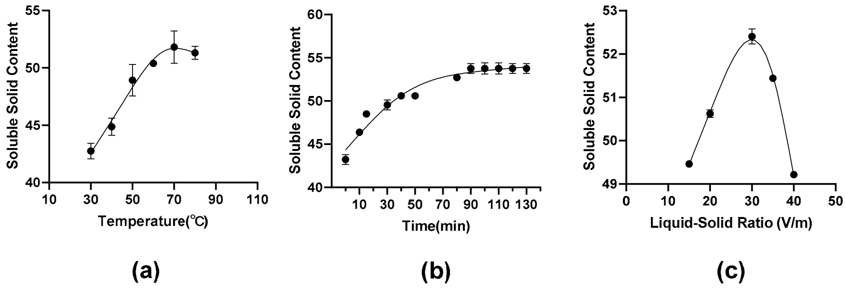

Figure S1. Influence of the time, temperature, and liquid-solid ratio on soluble solid extraction from Se-rich green tea. (**a**) Treatment conditions: liquid-solid ratio of 20 (v/m) for 30min extraction., (**b**) Treatment conditions: liquid-solid ratio of 20 (V/m) at 30℃ extraction., (**c**) Treatment conditions: extraction 30min at 30℃.

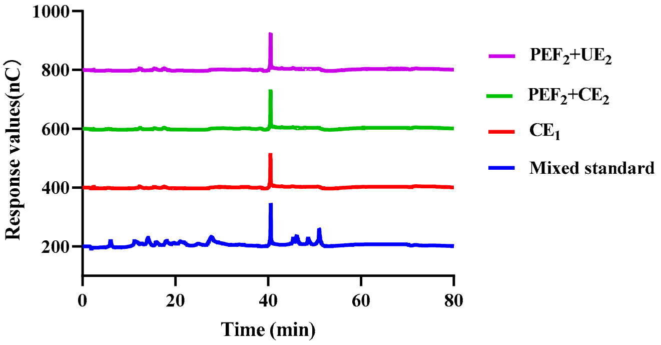

Figure S2. The ion chromatograms of Se-TPS obtained by different extraction methods.

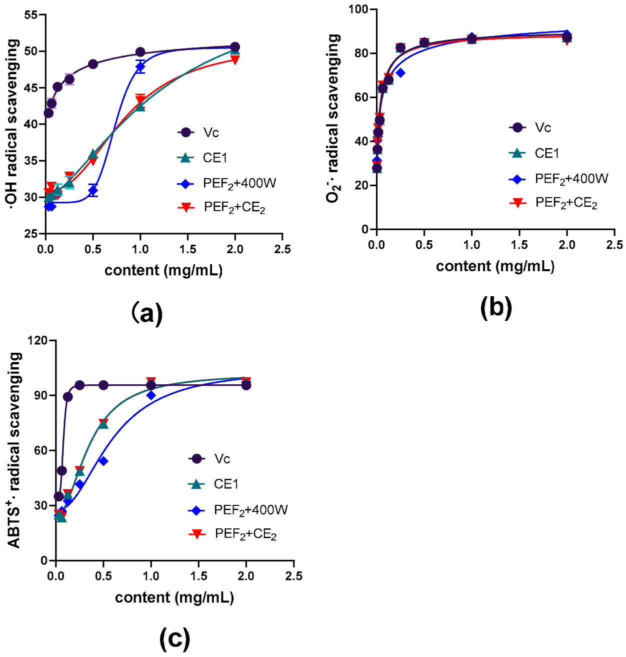

Figure S3 Antioxidant capacity of TPS from CE_1, PEF_2+CE_2, and PEF_2+UE_2. (**a**) ·OH assay, (**b**) $O_2^- \cdot$ assay, (**c**) $ABTS^+ \cdot$ assay.

Table S1. The detailed information for TPS extraction tests.

Text	CE_1	CE_2	CE_3	UE_1	UE_2	UE_3	PEF_1	PEF_2
Temperature	30℃	50℃	70℃	30℃	30 ℃	30 ℃	30 ℃	30 ℃
Power	/	/	/	100W	400 W	700 W	/	/
electric field intensity	/	/	/	/	/	/	4k V/cm	10 kV/cm
Time	60 min	60 min	60 min	60 min	60 min	60 min	1.80 ms	1.80 ms
solid-liquid ratio	1:30	1:30	1:30	1:30	1:30	1:30	1:30	1:30

Table S2. The basic ingredient of selenium-rich tea

Samples	Water content (%)	Aqueous extract (%)	Tea protein (%)	Tea polyphenol (%)	Caffeine (%)	Free amino acid (%)	Total sugar (%)	Total selenium (mg/kg)
Se-enriched tea	5.28±0.02	47.88±0.57	28.70±0.60	22.61±0.46	4.36±0.11	4.97±0.09	13.69±0.88	2.16±0.08

Data are means ± standard deviation, n = 3 independent experiments with 3 replicates in each experiment.

Table S3. The correlation between chemical composition and antioxidative activity

	TS	ABTS	OH	O^{2-}	TPP	TP	UA	NS	RS
TS	1	0.794**	0.816**	0.596**	0.878**	0.883**	0.791**	0.856**	0.878**
ABTS	0.794**	1	0.924**	0.860**	0.895**	0.893**	0.877**	0.871**	0.893**
OH	0.816**	0.924**	1	0.705**	0.949**	0.943**	0.933**	0.941**	0.942**
O^{2-}	0.596**	0.860**	0.705**	1	0.692**	0.689**	0.681**	0.684**	0.688**
TPP	0.878**	0.895**	0.949**	0.692**	1	0.999**	0.985**	0.965**	0.999**
TP	0.883**	0.893**	0.943**	0.689**	0.999**	1	0.986**	0.953**	10.00**
UA	0.791**	0.877**	0.933**	0.681**	0.985**	0.986**	1	0.929**	0.987**
NS	0.856**	0.871**	0.941**	0.684**	0.965**	0.953**	0.929**	1	0.951**
RS	0.878**	0.893**	0.942**	0.688**	0.999**	1**	0.987**	0.951**	1

** indicates significant correlation (P<0.01).
